# MiR-137 Targets the 3′ Untranslated Region of *MSH2*: Potential Implications in Lynch Syndrome-Related Colorectal Cancer

**DOI:** 10.3390/cancers13184662

**Published:** 2021-09-17

**Authors:** Raffaella Liccardo, Raffaele Sessa, Silvia Trombetti, Marina De Rosa, Paola Izzo, Michela Grosso, Francesca Duraturo

**Affiliations:** Dipartimento di Medicina Molecolare e Biotecnologie Mediche, Università di Napoli Federico II, 80131 Naples, Italy; raffaella.liccardo@unina.it (R.L.); raffaele.sessa@unina.it (R.S.); silvia.trombetti@unina.it (S.T.); marina.derosa@unina.it (M.D.R.); paola.izzo@unina.it (P.I.); michela.grosso@unina.it (M.G.)

**Keywords:** miR137, *MSH2*, Lynch Syndrome, regulation *MSH2* mRNA, 3′UTR MSH2, *MMR* genes

## Abstract

**Simple Summary:**

In a previous study, we identified a patient carrying the variant c.*226A>G in the *MSH2* 3′ untranslated region that showed overexpression of MSH2 protein. In this study, we hypothesized that the *MSH2* 3′UTR contained a seed region for miR-137, indicating *MSH2* was indeed targeted by miR-137. Our in vitro studies showed an inverse correlation between miR-137 and *MSH2*; this functional interaction was also confirmed in normal and tumoral tissue samples from the patient carrying the c.*226A>G variant in the *MSH2* 3′UTR.

**Abstract:**

Mismatch Repair (*MMR*) gene dysregulation plays a fundamental role in Lynch Syndrome (LS) pathogenesis, a form of hereditary colorectal cancer. Loss or overexpression of key *MMR* genes leads to genome instability and tumorigenesis; however, the mechanisms controlling *MMR* gene expression are unknown. One such gene, *MSH2*, exerts an important role, not only in MMR, but also in cell proliferation, apoptosis, and cell cycle control. In this study, we explored the functions and underlying molecular mechanisms of increased *MSH2* expression related to a c.*226A>G variant in the 3′untranslated (UTR) region of *MSH2* that had been previously identified in a subject clinically suspected of LS. Bioinformatics identified a putative binding site for miR-137 in this region. To verify miRNA targeting specificity, we performed luciferase gene reporter assays using a *MSH2* 3′UTR psiCHECK-2 vector in human SW480 cells over-expressing miR-137, which showed a drastic reduction in luciferase activity (*p* > 0.0001). This effect was abolished by site-directed mutagenesis of the putative miR-137 seed site. Moreover, in these cells we observed that miR-137 levels were inversely correlated with *MSH2* expression levels. These results were confirmed by results in normal and tumoral tissues from the patient carrying the 3′UTR c.*226A>G variant in *MSH2.* Finally, miR-137 overexpression in SW480 cells significantly suppressed cell proliferation in a time- and dose-dependent manner (*p* < 0.0001), supporting a role for MSH2 in apoptosis and cell proliferation processes. Our findings suggest miR-137 helps control *MSH2* expression via its 3′UTR and that dysregulation of this mechanism appears to promote tumorigenesis in colon cells.

## 1. Introduction

Mismatch repair (MMR) deficiency is derived from loss of function of MMR proteins. Approximately 10–15% of all colorectal cancers (CRCs) are MMR-deficient [1]. At the somatic level, this condition determines microsatellite instability (MSI) [2,3,4,5]. In some instances, MMR deficiency is associated with *MLH1* promoter hypermethylation which is responsible for sporadic CRC with MSI [6,7], whereas on other occasions, it correlates with germline pathogenic variants in *MMR* genes responsible for the Lynch Syndrome (LS) phenotype [3,4,5,8]. *MSH2* and *MLH1,* but also *MSH6* and *PMS2* are the most common pathogenetic variants [2,3,4,9,10,11,12] determining MMR deficiency in tumoral tissue; this is due to altered MMR protein functionality. However, it is accepted that in addition to canonical roles in MMR, the MMR pathway is also involved in a signaling cascade leading to cell cycle arrest and/or apoptosis [13,14,15,16] in the presence of DNA damage. Therefore, if the MMR system is proficient in cells, DNA damage levels may stimulate the system to inhibit the cell cycle or stimulate apoptosis [14]. Additionally, if MMR deficiency occurs in cells, the cell cycle does not block or activate apoptosis facilitating uncontrollable net cell division increases, therefore contributing to an increased mutation rate. This becomes manifest as MSI and/or abnormal MMR as shown by immunohistochemistry [16]. However, these MMR protein-driven mechanisms remain unclear [17,18]. To date, several hypotheses purporting to account for loss of function of the MMR system have been postulated. For example, etiological factors such as microRNAs (miRNAs) strongly influence repair functions within the MMR complex, with significant effects on disease progression [8,19,20,21,22]. MiRNAs constitute a family of small, single-stranded endogenous, non-coding, RNAs of approximately 21–25 nucleotides, the fundamental role of which is to negatively regulate gene expression at post-transcriptional levels. Individual miRNAs may be considered to be small control elements contributing to complex pathways underlying many fundamental functions, including cell cycle regulation, cell proliferation, differentiation, and apoptosis [23]. However, it is unclear if alterations in miRNA expression levels at tumor tissue are the cause or consequence of processes directly involved in tumorigenesis [24,25]. For example, a single nucleotide substitution in the miRNA ‘seed’ region (isomiR), or variants in messenger RNA target sites, can create or abolish miRNA interactions with molecular targets, and therefore induce expression variations in corresponding miRNAs or variations in target gene expression [19,22,26].

Several studies have described miRNA associations with colorectal tumorigenesis, with a possible role for miRNAs in *MLH1* regulatory expression [22,27,28].

Recently, we described a patient case where *MSH2* overexpression was likely related to a loss of *MSH2* down-regulation due to a nucleotide variant in the 3′UTR [8]. Preliminary bioinformatics analyses showed this region was a putative target site for miRNAs. Therefore, we investigated if the *MSH2* 3′UTR was a target site for miRNA-137, and examined if *MSH2* underwent post-transcriptional regulation by miR-137.

## 2. Materials and Methods

### 2.1. Bioinformatic Analysis

For the identification of putative miRNA target sites, TargetScan 6.2 (http://www.targetscan.org, accessed on June 2012) and miRanda 3.3 (http://www.microrna.org, accessed on August 2010) software were used.

### 2.2. Luciferase Constructs and Reporter Assay

**Figure 1:**
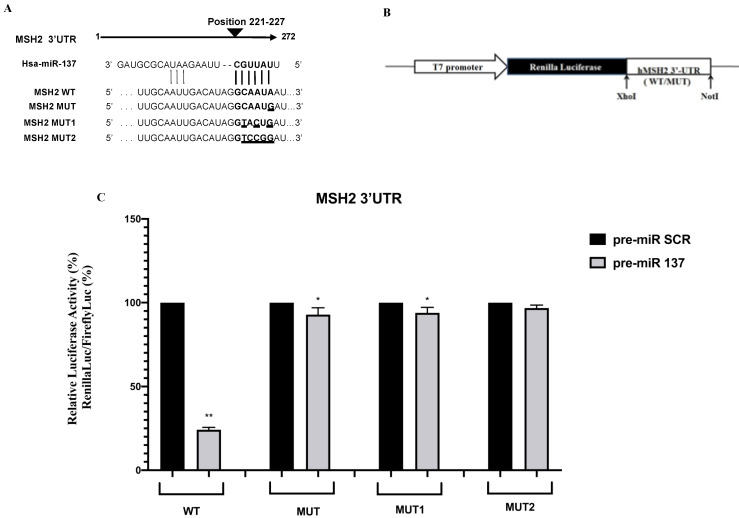
Luciferase *MSH2* 3′UTR constructs and luciferase reporter assay. (**A**) Wild-type (WT) and mutant *MSH2* 3′UTR reporter constructs are shown, aligned with the native miR-137 sequence. *MSH2* target sequences complementary to the miR-137 seed region are shown in bold and mutated bases are underlined. (**B**) Schematic representation of luciferase reporter gene construct. Constructs were cloned into the PSICHECK2 vector (Materials and Methods). (**C**) Reporter luciferase activity in WT and mutated (MUT, MUT1, MUT2) *MSH2* 3′UTR was measured in SW480 cells after transfection with pre-miR-137 and pre-miR scramble, (SCR) reagents. Data were normalized to Firefly luciferase activity. Values are expressed in percentages as the mean ± standard deviation (SD) of 4 samples assayed in 3 independent experiments (* *p* < 0.05; ** *p* < 0.0001).

The *MSH2* 3′ UTR of a carrier of the mutation c.*226 A>G was amplified by PCR with a primer pair containing *XhoI* and *NotI* restriction sites as already described. Oligonucleotide sequences were as follows: XhoI-3′ UTR *MSH2* forward: ATACTCGAGAAAATCCCAGTAATGGAATG and NotI-3′ UTR *MSH2* reverse: ATAGCGGCCGCTTCAAATTCCACAAACTACA. The PCR product was cloned into the PSICHECK2 vector (Promega, Madison, WI, USA) downstream of the *Renilla* luciferase coding region (hRluc). The orientation of the wild-type (WT) and mutated (MUT) inserted products was established by digestion and confirmed by sequencing. The PSICHECK2 constructs with additional mutations in the target site for miR-137 were generated using the QuiKChange Mutagenesis kit (Agilent Technologies, Santa Clara, CA, USA). Oligonucleotide sequences for site-directed mutagenesis were as follows: 3′UTR *MSH2* MUT1 forward, GGACTGTTTGCAATTGACATAGGTACTgATAAGTGATGTGCTG and reverse, CAGCACATCACTTATcAGTACCTATGTCAATTGCAAACAGTCC; 3′UTR *MSH2* MUT2 forward, GGACTGTTTGCAATTGACATAGGTCCGgATAAGTGATGTGCTG, and reverse, CAGCACATCACTTATcCGGACCTATGTCAATTGCAAACAGTCC (seed region is underscored, patient mutated base is in lowercase, and additional mutated bases are in bold, Figure 1A). Luciferase activity was measured 48 h after transfection using a dual luciferase reporter assay (Promega) according to manufacturer’s instructions and performed on a 20n/20n luminometer (Turner BioSystems, Sunnyvale, CA, USA). Relative luciferase activity was calculated by normalizing the *Renilla* luminescence to the firefly luminescence [29].

### 2.3. Cell Culture and Transfection

SW480 colorectal adenocarcinoma cells (*ATCC CCL-228*) were cultured in RPMI 1640 (Sigma-Aldrich, Milan, Italy) supplemented with 10% fetal bovine serum (Sigma-Aldrich), 1% GlutaMAX-I (Gibco Invitrogen Life Technologies, CA, USA), and 1% penicillin/streptomycin (Gibco Invitrogen Life Technologies). The cultures were maintained at 37 °C in a 5% CO_2_-humidified atmosphere and were kept sub-confluent (70–80%) for the subsequent transfection experiments. Twenty-four hours before transfection, adherent cells were plated into 96-well plates at a density of 25 × 10^3^ cells/well (70–90% confluence) in 100 µL of Optimem medium (Invitrogen Life Technologies) in the absence of serum/antibiotics. Transfections for luciferase assay were carried out using 30 ng of each PSICHECK2 construct with 30 pmol of miRNA precursor *hsa-miR-137* or relative FAM negative control (Ambion, Life Technologies, CA, USA).

Total endogenous RNA and protein were isolated from SW480 cells after transfection with 15 and 30 pmol pre-miR miRNA precursor *hsa-miR-137* or relative FAM pre-miR-negative control, as previously described [30,31]. Similarly, total endogenous RNA and protein were isolated from SW480 cells after transfection with 30, and 60 of anti-miR miRNA inhibitor *hsa-miR-137* or relative FAM negative control (Ambion, Life Technologies). Twenty-four hours before transfection, adherent cells were plated into 6-well plates at a density of 25 × 10^3^ cells/well (60–70% confluence) in 2 mL of Optimem medium (Invitrogen Life Technologies) in the absence of serum/antibiotics.

All transfections were performed using Lipofectamine 2000 (Invitrogen Life Technologies), according to the manufacturer’s instructions.

### 2.4. SW480 Total RNA and Protein Analysis

Total RNA and protein were extracted 48 h after miRNA and anti-miRNA transfection. RNA was isolated with TRIZOL solution according to the manufacturer’s protocols, and was used to determine endogenous mRNA levels of *MSH2* and *actin* (internal control) according to the same protocol reported for RNA analysis of patient carrying the variant in *MSH2* 3′UTR gene [8]. The dosage of *miR-137* levels was performed using a TaqMan MiRNA RT and Assay kit (Applied Biosystems, Inc.,Waltham, Massachusetts, Stati Uniti), with small nucleolar RNA *RNU6B* (Applied Biosystems, Inc.) as a normalization gene. Real-time PCR reactions were performed in triplicate.

Total protein was isolated using cell lysis buffer containing 10% glycerol, 50 mM Tris-HCl pH 8.0, 150 mM NaCl, 0.1% NP-40, 1 mM EDTA pH 8, and 10 µL of protease inhibitor cocktail (Sigma-Aldrich). Quantification of MSH2 and actin (loading control) was performed as described for the patient protein analysis [8].

### 2.5. Formalin-Fixed and Paraffin-Embedded (FFPE) Tissues RNA Isolation and Expression Assay

miRNA and mRNA were extracted from FFPE samples of colon tumor and normal tissue of patient carrying variant in 3′UTR of MSH2 gene using Qiagen miReasy FFPE Kit (Qiagen, Hilden, Germany) according to the manufacturer’s instructions. The RNA quantity was assessed using the Qubit RNA BR Assay Kit (Invitrogen, by Thermo Fisher Scientific, Eugene, Oregon, USA). The expression of miR-137 and *MSH2* as well as the relative endogenous controls, U6 snRNA and GAPDH, respectively, were determined using TaqMan MicroRNA Reverse Transcription Kit and specific TaqMan miRNA assays (Taq-Man, Applied Biosystems, Inc.,Waltham, Massachusetts, Stati Uniti). All qRT-PCR assays for normal and tumor sample were performed in triplicate using the same amount of isolated RNA.

### 2.6. Analysis of Cell Proliferation

Cell viability was determined using the MTT [3-(4,5-dimethylthiazol-2-yl)-2,5-diphenyltetrazolium bromide] assay. Briefly, after transient transfection with 15 and 30 pmol of pre-miR-137 and pre-miR negative control, SW480 cells were seeded into a 96 well plate at a concentration of 5 × 10^3^ cells/100 μL. At 24, 48, 72, 96, and 120 hrs after transfection, respectively, 10 μL of MTT labeling reagent provided by the Cell Proliferation Kit 1 (Roche, Mannheim, Germany) was added to each well as previously reported [32]. Measurement of the soluble formazan product in each well was carried out by photometric reading at 570/690 nm on a Synergy H1 Hybrid Multi-Mode Microplate Reader (BioTek, Winooski, VT). The experiments were repeated three times for each transfection. All data are reported as the mean ± standard deviation of three separate experiments. Statistical differences between mock control and treated cells were calculated using the one-way analysis of variance procedure followed by Dunnett’s multiple comparison test, where appropriate. Differences were considered significant when *p* < 0.05 (*) and highly significant when *p* < 0.0001 (**) versus each respective control or untreated control group.

### 2.7. Statistical Analysis

Data from luciferase assays, real-time quantitative PCR, and Western blot analysis were analyzed using GraphPad Prism 6.0 software (GraphPad Software, Inc., 2365 Northside Dr. Suite 560 San Diego, CA 92108). Mean values (±S.D.) were calculated. Statistical significance was determined using Student’s *t*-test. Data were considered statistically significant when *p*-value was <0.05 and highly significant when *p-* value was <0.0001.

## 3. Results

### 3.1. Identification of MiR-137 Using in Silico Analysis

In our previous study, we showed that a point mutation in the 3′UTR of *MSH2* (c.*226A>G) determined *MSH2* overexpression and was associated with LS [8]. Here, we performed further computational analyses using TargetScan 6.2 and miRanda 3.3 software to identify a putative binding site for miRNA to explain the regulatory loss of the *MSH2* transcript. In the *MSH2* 3′UTR, we identified a putative binding site for miRNA-137 which was altered by the presence of the c.*226A>G mutation, suggesting a possible role for miR-137 in negatively regulating *MSH2* expression.

### 3.2. Functional Effects of the MSH2 3′UTR Variant on Luciferase Reporter Expression

In this study, to confirm *MSH2* as a direct target of miR-137, WT *MSH2* 3′UTR luciferase plasmid and 3′UTR mutants were co-transfected with 30 pmol pre-miR-137 precursor and a relative Fluorescein Amidites (FAM)negative control. Relative luciferase activity was significantly reduced in the presence of pre-miR-137 (*p* < 0.0001) (Figure 1). Additionally, gene reporter assays indicated miRNA-mediated *MSH2* regulation occurring at the 3′UTR site. Only when the putative seed region for miR-137 (WT) was kept unmodified there was a reduction in reporter gene activity compared to the control (Figure 1C) This effect was abolished by site-directed mutagenesis in the putative miR-137 seed site, suggesting *MSH2* 3′ UTR (position 221–227) was the direct target of miR-137. Among mutants, we found a direct correlation between the number of mutated nucleotides within the seed region and the increased luciferase activity. In fact, the MUT and MUT1 constructs containing a partly mutated seed sequence (Figure 1A) showed a slight reduction in the luciferase activity (Figure 1C) whereas the MUT2 construct showed no variation in gene expression when compared to the control (Figure 1C).

### 3.3. MiR-137 Regulates MSH2 Expression

**Figure 2:**
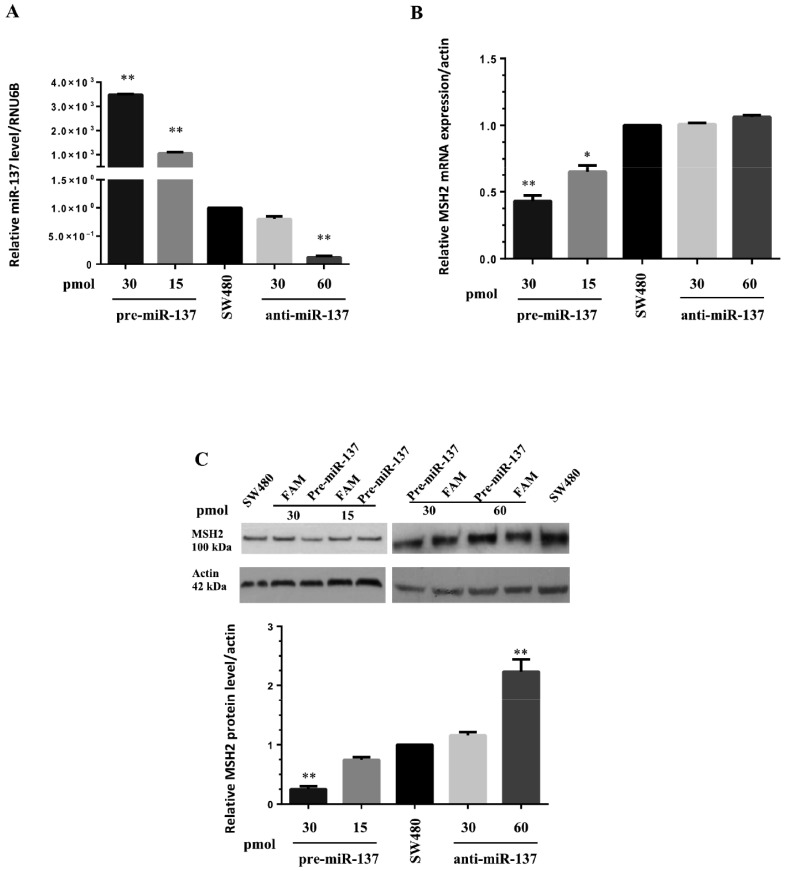
Transfection of pre-miR-137 and anti-miR-137 reagents into SW480 cells. (**A**) Transfected pre-miR-137 (15 and 30 pmol) and anti-miR-137 (30 and 60 pmol) levels were determined by qRT-PCR using RNA isolated from SW480 cells, normalized to small nucleolar RNA RNU6B, and compared with endogenous miR-137 expression levels. (**B**) *MSH2* mRNA levels in SW480 cells transfected with pre-miR-137 and anti-miR-137 by qRT-PCR, normalized against actin used as internal control. (**C**) MSH2 protein levels in SW480 cells transfected with pre-miR-137 and anti-miR-137 by Western blotting with densitometric analysis of MSH2 protein and mRNA expression, normalized to actin, compared to each FAM negative control and endogenous MSH2 expression. Data represent the mean ± standard deviation (SD) of 3 independent experiments. Statistical significance was determined by Student’s *t*-test with * *p* < 0.05 and ** *p* < 0.0001.

We transfected pre-miR-137 and anti-miR-137 into SW480 cells at different concentrations. As expected, pre-miR and anti-miR transfected into SW480 cells have determined increased and decreased miR-137 levels, respectively, when compared with not transfected cells (Figure 2). Thus, in these cells, we measured *MSH2* mRNA and protein levels. We showed that miR-137 overexpression was associated with MSH2 down-regulation at both mRNA and protein levels, suggesting ectopic miR-137 correlated with a significant decrease in these levels (Figure 2B,C). SW480 cells were also transfected with anti-miR-137 to demonstrate miR-137 specificity toward *MSH2*. In this scenario, blocking endogenous miR-137 activity with anti-miR-137 treatment increased mRNA and protein MSH2 production, confirming a negative regulatory role for miR-137 on MSH2 (Figure 2B,C).

### 3.4. MiR-137 and mRNA MSH2 Levels Are Inversely Correlated in a Patient Carrying a 3′UTR Variant, the c.*226A>G

**Figure 3:**
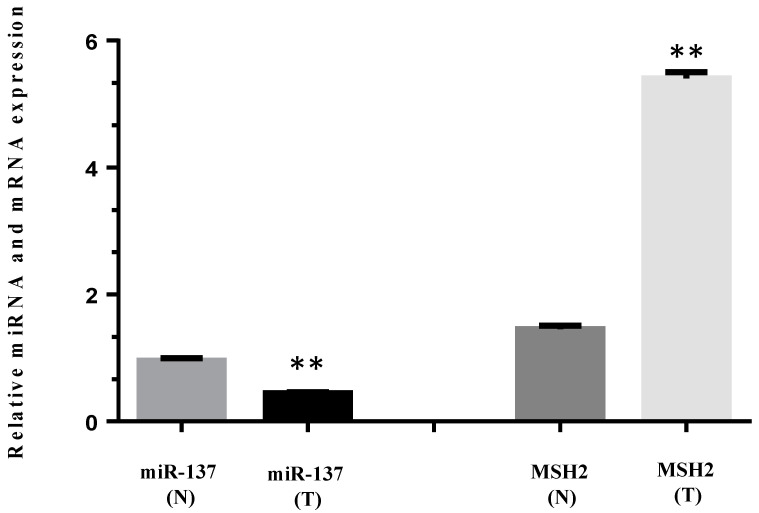
Relative pre-miR-137 and *MSH2* mRNA expression in normal (N) and tumor (T) colon tissue from a patient carrying the c*226>G variant in the *MSH2* 3′UTR. Pre-miR-137 levels were determined by qRT-PCR and normalized to small nucleolar RNA RNU6B. *MSH2* mRNA levels were normalized to *GAPDH*. All data represent the mean ± standard deviation (SD) of 4 independent experiments. Statistical significance was determined by Student’s *t*-test with a ** *p* < 0.0001.

We determined the relative expression of miRNA-137 and *MSH2* mRNA levels in normal and tumoral colon sample from a patient carrying the 3′UTR *MSH2* variant, the c.*226A>G. Tumor tissue (T) showed significantly reduced miR-137 expression when compared with the normal section (N), while significant *MSH2* overexpression was observed in the tumor versus the normal sample (Figure 3).

### 3.5. MiR-137 Effects on SW480 Cell Proliferation

**Figure 4:**
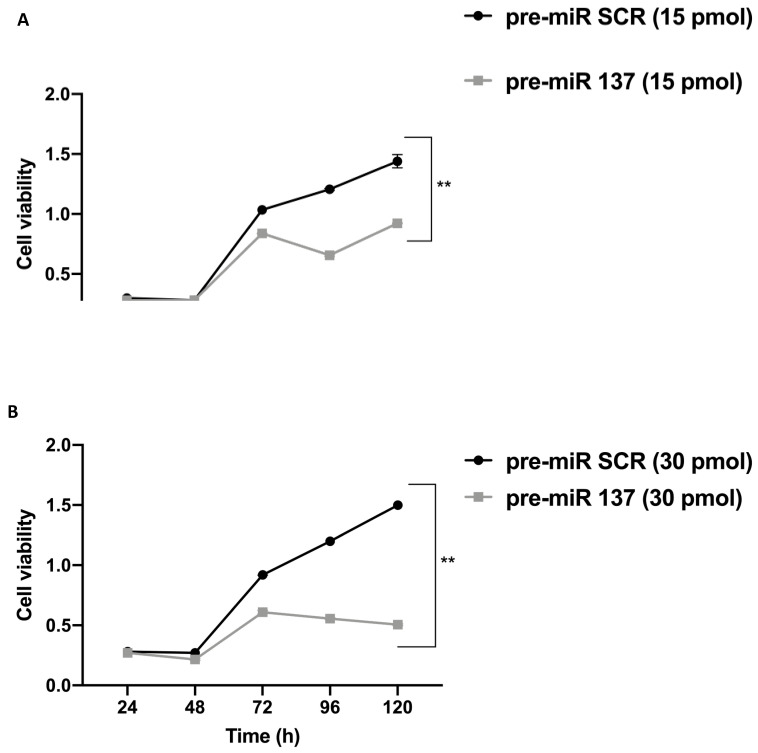
A 3-(4,5-dimethylthiazol-2-yl)-2,5-diphenyl-2H-tetrazolium bromide (MTT) assays was used to determine cell viability in SW480 cells transfected with 15 (**A**) and 30 (**B**) pmol pre-miR-137 and pre-miR SCR (negative control), from 24–120 h. MiR-137 overexpression significantly suppressed SW480 proliferation in a time- and dose-dependent manner (** *p* < 0.0001).

A 3-(4,5-dimethylthiazol-2-yl)-2,5-diphenyl-2H-tetrazolium bromide (MTT) assay was performed on SW480 cells transfected with pre-miR-137 and pre-miR scramble (SCR) and showed that miR-137 overexpression significantly suppressed cell proliferation in a time- and dose-dependent manner (** *p* < 0.0001). In particular, at 72 h post-transfection with pre-miR-137, progressively reduced cell proliferation, even when compared with the pre-miR SCR counterpart (Figure 4), was observed. These data suggested a potential anti-proliferative role for miR-137 in these cells.

## 4. Discussion

We investigated the increased *MSH2* expression, previously identified in an individual clinically suspected with LS, and carrying a 3′UTR region sequence variant. Our data suggested miR-137 controlled *MSH2* expression via the 3′UTR, and its dysregulation putatively promoted tumorigenesis in colon cells. Recently, Zhou et al. reported the role of miR-137 in LS tumor pathogenesis [33], potentially reinforcing our hypothesis that *MSH2* is targeted by miR-137.

Several reports have suggested that *MMR* cellular levels are tightly regulated [21,22,24]. In addition to the well-characterized oncogenic events such as genetic amplification, mutation, and translocation, *MMR* dysregulation also leads to loss of the MMR system [34,35,36]. Bioinformatics analyses predicted the presence of a miR-137 seed region in the *MSH2* 3′UTR—with our in vitro results confirming *MSH2* is targeted by miR-137. MiR-137, which is located on human chromosome 1p22, is reportedly present in neuronal cells [37,38,39,40], but is also found constitutively expressed in normal colonic epithelium [27,28,41,42,43,44], while low expression is found in adenomatous polyps and tumoral colorectal tissues [21]. Moreover, miR-137 locus is frequently hypermethylated in several tumor types, including colorectal, gastric, breast, head and neck squamous cell carcinoma, and colorectal adenomatous tissue [45,46,47,48]. It was also reported that miR-137 locus hypermethylation was associated with MSI [48,49,50]. Previous data indicated that average methylation levels of miR-137 locus were much higher (approximately 80%) in most colon cell lines, including HCT116, RKO, SW48, and HT29, but not LoVo and SW480 cells where miR-137 methylation was found around 28–29%, respectively [48]. However, LoVo cells are constitutively MSH2-deficient as well established in the literature [51,52] and, therefore unsuitable for the aim of this study. Based on the lower methylation status of miR-137 locus, only the SW480 cell system was found to be suitable for our expression studies with respect to other colon cancer cell lines commercially available, to better evaluate the correlation between miR-137 and its potential target gene, *MSH2* in the specific case.

Transient transfection of SW480 cells with a WT reporter plasmid and a miR-137 precursor decreased luciferase activity that remained unaffected following transfection with the mutated 3′UTR constructs, as reported in Figure 1. In our previous study [8] beyond a higher luciferase activity than in the construct with the WT 3′ UTR, a greater luciferase expression was observed in proportion to the number of additional mutations created by in vitro mutagenesis. Therefore, we hypothesized that the *MSH2* 3′UTR contained a seed region for miR-137, indicating *MSH2* was indeed targeted by miR-137. Additionally, our in vitro studies showed an inverse correlation between miR-137 and *MSH2* in SW480 cells; this functional interaction was also confirmed in normal and tumoral tissue samples from patient carrying the c.*226A>G variant in the *MSH2* 3′UTR. In a previous study, in this patient, *MSH2* was found to be overexpressed at the somatic level, as confirmed by immunohistochemistry [8]. In the current study, we confirmed this result also on mRNA extracted from the tumoral tissue. Moreover, we observed a reduced expression of miR-137, likely associated with hypermethylation of the miR-137 locus, as described in tumor tissues (colorectal adenoma and adenocarcinoma) and in most of the colon cancer cells lines commercially available [48]. However, also molecular alterations that create or abolish miRNA interactions with molecular targets could determine expression variations in corresponding miRNAs [19,22,26]. Therefore, we propose miR-137 negatively regulates *MSH2* expression at the post-transcriptional level. Although the use of a single cell line may have limited our study outcomes, we believe that using other CRC cell lines may have provided misleading results due to the high degree of miR-137 methylation potentially masking this inverse relationship between miR-137 and *MSH2*. Furthermore, we directly validated this result using normal and tumor tissue derived from the patient that in our opinion could represent a more physiologically relevant setting to corroborate our findings. Another possible study limitation was that we could not confirm our observations in other patients carrying this variant in *MSH2* 3′UTR because no other family members were available for this study. In our patient, this inverse correlational relationship could be due to sequence variants preventing miR-137 binding to *MSH2*; meaning *MSH2* regulation is lost. Increased *MSH2* expression could result in a failure to form a functioning heterodimer (MUTS), generating an unstable phenotype during LS tumorigenesis [20]. However, increased *MSH2* mRNA could also directly favor the tumorigenic process. Several studies reported that the MSH2 protein was involved in apoptosis and cell proliferation [53]. It is accepted that MutSα (MSH2-MSH6 heterodimer) and MutLα (MLH1-PMS2 heterodimer) directly recruit ATM/ATR to induce cell cycle arrest and/or apoptosis [54] and that loss of MMR function system increases mutation frequency and is associated with tumorigenesis [15]. Therefore, dysregulated *MSH2* expression could prevent apoptosis and favor a tumorigenic state. In this respect, our cell viability data confirmed the suppressor role of miR-137 toward SW480 proliferation, in agreement with MSH2 functions in apoptotic and tumorigenesis processes. Thus, we hypothesize that MSH2 overexpression, due to a loss of miR-137 targeting and aberrant MUTS heterodimer formation, could interfere with cell cycle proteins to promote tumorigenesis. Therefore, following miR-137 methylation in CRC cell lines and tissue [48], we speculate *MSH2* loses its regulatory capabilities resulting in increased genomic instability and tumorigenesis. Although further studies are required to corroborate our observations, we hypothesize miR-137 putatively acts as a *MSH2* regulator via its 3′UTR.

In conclusion, our data suggest miR-137 targets the 3′UTR region of *MSH2* mRNA and directly down-regulates *MSH2* expression. Moreover, our study highlighted the important role of miRNAs as regulators of *MMR* gene expression.

Further investigations are required to identify the potential prognostic and therapeutic implications of miR-137 in patients with LS to provide precision medicines for this pathogenic condition [55].

## 5. Conclusions

In conclusion, this study has provided a new insight on the molecular mechanisms that regulate the MMR gene products. Our work has highlighted the important role of miRNA molecules as likely regulators of MMR gene expression, whose effects are often mediated through binding with 3′ UTRs. To our best knowledge, this is the first report indicating that miR-137 regulates MSH2 expression. Moreover, we have confirmed the widely reported tumor-suppressive function of miR-137 that demonstrated its implication in LS as a negative regulator of MSH2. Further investigations will be required to identify the potential prognostic and therapeutic implications of miR-137 in LS patients.

## Data Availability

The data presented in this study are available on request from the corresponding author.
